# Translational Chickpea Genomics Consortium to Accelerate Genetic Gains in Chickpea (*Cicer arietinum* L.)

**DOI:** 10.3390/plants10122583

**Published:** 2021-11-25

**Authors:** Ramesh Palakurthi, Veera Jayalakshmi, Yogesh Kumar, Pawan Kulwal, Mohammad Yasin, Nandkumar Surendra Kute, Chinchole Laxuman, Sharanabasappa Yeri, Anilkumar Vemula, Abhishek Rathore, Srinivasan Samineni, Khela Ram Soren, Biswajit Mondal, Girish Prasad Dixit, Chellapilla Bharadwaj, Sushil K. Chaturvedi, Pooran M. Gaur, Manish Roorkiwal, Mahendar Thudi, Narendra P. Singh, Rajeev K. Varshney

**Affiliations:** 1 International Crops Research Institute for the Semi-Arid Tropics (ICRISAT), Patancheru 502324, India; palakurthiramesh1@gmail.com (R.P.); anil.kumar@cgiar.org (A.V.); a.rathore@cgiar.org (A.R.); s.srinivasan@cgiar.org (S.S.); p.gaur@cgiar.org (P.M.G.); manishroorkiwal@gmail.com (M.R.); 2 Regional Agricultural Research Station (RARS), Acharya N.G. Ranga Agricultural University (ANGRAU), Nandyal 518501, India; veera.jayalakshmi@gmail.com; 3 ICAR-Indian Institute of Pulses Research (IIPR), Kanpur 208024, India; yogeshtiwari70@gmail.com (Y.K.); sorenars@gmail.com (K.R.S.); biswagpb@gmail.com (B.M.); gpdixit1966@rediffmail.com (G.P.D.); sushil.chaturvedi@gmail.com (S.K.C.); 4 Department Agricultural Botany, Mahatma Phule Krishi Vidyapeeth (MPKV), Rahuri 413722, India; pawankulwal@gmail.com (P.K.); nskute2019@gmail.com (N.S.K.); 5 RAK College of Agriculture (RAKCA), Rajmata Vijayaraje Scindia Krishi Vishwa Vidyalaya (RVSKVV), Sehore 466001, India; myasin23@gmail.com; 6 Agricultural Research Station (ARS), University of Agricultural Sciences (UAS), Raichur 584104, India; laxumanc@uasraichur.edu.in (C.L.); sbyeri@uasraichur.edu.in (S.Y.); 7 Division of Genetics, ICAR-Indian Agricultural Research Institute (IARI), Delhi 110012, India; drchbharadwaj.iari@gmail.com; 8 College of Agriculture, Rani Lakshmi Bai Central Agricultural University, Jhansi 284003, India; 9 Department of Agricultural Biotechnology and Molecular Biology, Dr. Rajendra Prasad Central Agricultural University (RPCAU), Pusa 848125, India; 10 State Agricultural Biotechnology Centre, Centre for Crop and Food Innovation, Food Futures Institute, Murdoch University, Murdoch, WA 6150, Australia

**Keywords:** chickpea, marker assisted backcross, farmer participatory varietal selection, multi-location trials, drought, Fusarium wilt

## Abstract

The Translational Chickpea Genomics Consortium (TCGC) was set up to increase the production and productivity of chickpea (*Cicer arietinum* L.). It represents research institutes from six major chickpea growing states (Madhya Pradesh, Maharashtra, Andhra Pradesh, Telangana, Karnataka and Uttar Pradesh) of India. The TCGC team has been engaged in deploying modern genomics approaches in breeding and popularizing improved varieties in farmers’ fields across the states. Using marker-assisted backcrossing, introgression lines with enhanced drought tolerance and fusarium wilt resistance have been developed in the genetic background of 10 elite varieties of chickpea. Multi-location evaluation of 100 improved lines (70 desi and 30 kabuli) during 2016–2017 and 2018–2019 enabled the identification of top performing desi and kabuli lines. In total, 909 Farmer Participatory Varietal Selection trials were conducted in 158 villages in 16 districts of the five states, during 2017–2018, 2018–2019, and 2019–2020, involving 16 improved varieties. New molecular breeding lines developed in different genetic backgrounds are potential candidates for national trials under the ICAR-All India Coordinated Research Project on Chickpea. The comprehensive efforts of TCGC resulted in the development and adoption of high-yielding varieties that will increase chickpea productivity and the profitability of chickpea growing farmers.

## 1. Introduction

Chickpea (*Cicer arietinum* L.; 2*x* = 2*n* = 16) is an important food legume crop cultivated on 13.72 M ha with a total production of 14.25 M t [1]. Although India is the largest producer of chickpea, it imports large quantities from Australia (83.5%), USA (3.8%), Myanmar (3.5%), Tanzania (3.3%), and Sudan (2.1%) to meet local demand (http://agricoop.nic.in/sites/default/files/Pulses%20profie_Mar%2C%202019_0.pdf; Last accessed on 25 July 2021). In terms of production in India, Madhya Pradesh ranks first, contributing 33.99% of the area and 40.92% of production, followed by Maharashtra, Rajasthan, Karnataka, Andhra Pradesh and Uttar Pradesh (http://dpd.gov.in/Annual%20Report%202017-18.pdf; Last accessed on 25 July 2021). Limited genetic diversity coupled with climate change during recent years has increased the frequency and severity of biotic and abiotic stresses and emerging diseases that are serious threats to chickpea production [2,3,4]. In order to achieve the crop’s actual yield potential, it is essential to enhance its genetic diversity and resistance/tolerance to biotic and abiotic stresses in the varieties grown by farmers.

Modern breeding technologies have proven useful in developing superior varieties in crops such as maize, rice, wheat, barley and soybean [5]. This was not the case in chickpea until recently, primarily due to limited information on genes and the ability to deploy genomics tools. In recent years, the tremendous progress made in developing novel genomic tools in chickpea, such as the draft genome sequence [6], several millions of SNP markers from whole genome sequence information on germplasm lines [7,8,9] and cost-effective genotyping platforms including low- to high-density SNP arrays [10,11]. Likewise, QTLs and markers associated with abiotic stresses like drought [12,13,14,15], heat [16] and salinity [17], and biotic stresses like fusarium wilt (FW) [18,19] and Ascochyta blight (AB) [18,20] are available for chickpea improvement. These have facilitated marker-assisted selection/introgression in chickpea breeding programs. Marker-assisted backcrossing (MABC) approach has been successfully deployed to develop superior varieties in the crop [21].

Once the chickpea genome sequence was available, the Department of Agriculture & Farmers Welfare (DA & FW) encouraged a consortium approach to translate this knowledge to enhance chickpea improvement through funding support. As a result, the Translational Chickpea Genomics Consortium (TCGC) was established in 2016, comprising six research institutions/agricultural universities from six major chickpea growing states of India: International Crops Research Institute for the Semi-Arid Tropics (ICRISAT), Hyderabad, Telangana; Regional Agricultural Research Station (RARS), Nandyal, Andhra Pradesh; Agricultural Research Station (ARS), Kalaburagi, University of Agricultural Sciences (UAS), Raichur, Karnataka; RAK College of Agriculture (RAKCA), Rajmata Vijayaraje Scindia Krishi Vishwa Vidyalaya (RVSKVV), Sehore, Madhya Pradesh; Mahatma Phule Krishi Vidyapeeth (MPKV), Rahuri, Maharashtra; and Indian Institute of Pulses Research (ICAR-IIPR), Kanpur, Uttar Pradesh. These research institutions are very well catering needs of the almost 80% of the chickpea area in India. The consortium’s major focus has been on deploying genomics information for chickpea improvement, developing/identifying improved varieties and enhancing the adoption of superior lines in farmers’ fields. During the past five years, TCGC has made significant progress that has involved: (a) deployment of genomics-assisted breeding in 10 chickpea varieties for drought tolerance and fusarium wilt resistance; (b) evaluation of 100 improved elite breeding lines (70 desi and 30 kabuli) for their performance in multi-location trials and (c) demonstration of improved crop varieties in 909 farmers’ fields across 158 villages spanning 16 districts in the states of Andhra Pradesh, Karnataka, Madhya Pradesh, Maharashtra and Uttar Pradesh. This paper reports on the significant achievements of the TCGC that can guide future chickpea improvement programs and lead to the development and popularization of improved varieties in important regions where the crop is grown in India.

## 2. Materials and Methods

### 2.1. Marker-Assisted Backcrossing for Drought Tolerance and Fusarium wilt Resistance

**Figure 1:**
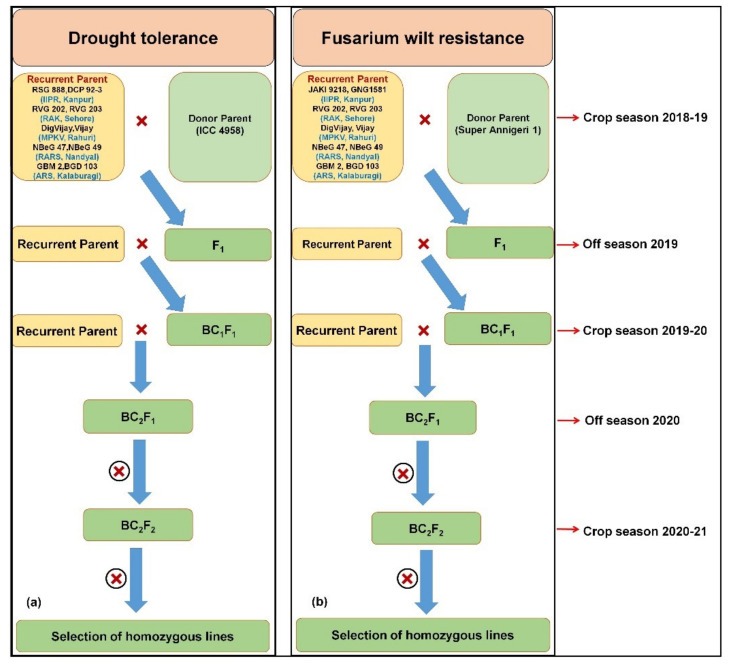
Marker-assisted backcrossing was adopted for (**a**) drought tolerance and (**b**) fusarium wilt resistance using two recurrent parents from each center. ICC 4958 was used to introgress “*QTL-hotspot*” harbouring QTLs for drought tolerance related traits and Super Annigeri 1 was used as donor to introgress FW resistance in elite chickpea cultivars.

The MABC approach was adopted to enhance drought tolerance and FW resistance in two leading varieties from each of the consortium partners (Figure 1). In brief, 10 select recurrent parents, namely RSG 888, DCP 92-3, RVG 202, RVG 203, DigVijay, Phule Vikram, NBeG 47, NBeG 49, GBM 2 and BGD 103 were crossed with ICC 4958 as a donor (*“QTL-hotspot”*) for drought tolerance (Figure 1). Similarly, the genomic regions for FW resistance were also introgressed into the genetic background of 10 elite varieties (JAKI 9218, GNG 1581, RVG 202, RVG 203, Digvijay, Vijay, NBeG 47, NBeG 49, GBM 2 and BGD 103) using Super Annigeri 1 as a donor (Figure 1).

### 2.2. Development of Trait-Associated Markers

To develop trait associated markers, available genomic information on candidate regions for drought and FW was collected from previous studies and focused on the identification of major QTL regions [12,14,15,18,19,20]. For drought, a total of 26 genes identified in the “*QTL-hotspot*” region containing 24 SNPs were used to develop molecular markers for use in early generation selection. As a result, 10 SNP panel for drought was developed and tested for early generation selection for the backcross progenies (Table 1). Similarly, for the development of FW markers, genomic information on candidate regions of QTL associated with FW resistance was assembled from the studies [18,19,20] (Table 1 and Table S1). We used these markers on the backcross progenies and validated their usefulness in chickpea breeding programs. These findings have been considered important milestones for accelerated chickpea breeding programs.

### 2.3. Multi-Location Trials for Promising Chickpea Lines

To identify the best performing lines across locations, 70 desi and 30 kabuli lines (including molecular breeding lines) were evaluated for their yield performance (kg/ha) during 2016–2017 and 2018–2019. Based on the availability of seed, multi-location trials were conducted in three locations (Jabalpur, Nandyal and Sehore) during 2016–2017 and in five locations (Nandyal, Kalaburagi, Sehore, Rahuri and Kanpur) during 2018–2019. However, kabuli lines were not evaluated in Sehore and Kanpur locations during 2018–2019 owing to limited seed availability. Desi and kabuli lines were evaluated in an alpha lattice design with 14 entries per block. A spacing of 30 cm × 10 cm (intra-row) and 4 m (inter-row) was adopted to evaluate desi lines and a spacing of 45 cm × 10 cm (intra-row) and 4 m (inter-row) was adopted for kabuli lines. Combined analysis of variance (ANOVA) was carried out using SAS mixed procedure [22] to test the significance of main and interaction effects of environments and genotypes, considering environment, genotype, replication and block as random effects. Individual environmental variances were modelled into combined analysis with REPEATED statement using REML (Restricted Maximum Likelihood) method. Best Linear Unbiased Predictors (BLUPs) were calculated from the combined analysis. To identify the best test environment and superior genotype with high yield and stable performance across locations, GGE (genotype (G) + (genotype (G) × environment (E) interaction), a biplot analysis was done with the mean grain yield data obtained from multi-location trials. Using SREG (Site regression), GGE biplots [23] were performed for significant interaction effects of district and variety (FPSV trials) and environment and genotype (desi and kabuli multi-environment trials) to draw conclusions on genotype and environment evaluations.

### 2.4. Varietal Adoption through Farmer Participatory Varietal Selection (FPVS) Trials

To enhance the adoption of improved varieties that are already available, FPVS trials were conducted in five major chickpea growing states (Andhra Pradesh, Karnataka, Madhya Pradesh, Maharashtra and Uttar Pradesh) in India during 2017–2018, 2018–2019 and 2019–2020 (Table 2). In total, 909 FPVS trials were conducted during three years. To date, the data from the three years has been compiled and analyzed. During the three years, two to five improved varieties in each state were distributed to farmers to conduct FPVS. A total of 909 FPVS trials were conducted and 16 improved chickpea varieties (two to five varieties in each state) were tested in 16 districts in the five states (Table 2).

## 3. Results

### 3.1. Genomics-Assisted Breeding

Drought and FW are major abiotic and biotic stresses that hamper chickpea production. Some of the chickpea varieties with enhanced drought tolerance and FW resistance which were developed earlier using MABC approach and released (https://icar.org.in/content/development-two-superior-chickpea-varieties-genomics-assisted-breeding; https://icar.org.in/content/development-two-superior-chickpea-varieties-genomics-assisted-breeding; last accessed on 25 July 2021) [24,25,26,27] are already in the seed chain and have started paying dividends.

F_1_s were harvested at each center by crossing chosen recipient parents with ICC 4958 as a donor (“*QTL-hotspot*”) for drought tolerance and with Super Annigeri 1 as a donor for FW resistance during 2018–2019. True hybrids were confirmed using 10 SNP panel and two allele specific markers for the respective traits (Table 1 and Table S1 in Supplementary Materials). Subsequently, BC_1_F_1_ were obtained by crossing true F_1_s to their respective recurrent parents during 2019–2020. At all centers, heterozygous plants were used for subsequent backcrossing with respective recurrent parents, and BC_2_F_1_ seeds were harvested during the off season 2020. After foreground selection, positive BC_2_F_1_ plants were selfed to generate BC_2_F_2_ seeds during crop season 2020–2021 (Table S2). The back cross progenies after background selection needs further evaluation for their yield performance under drought.

### 3.2. Mean Performance and Stability of Elite Breeding Lines for Grain Yield in Multi-Location Trials

**Figure 2:**
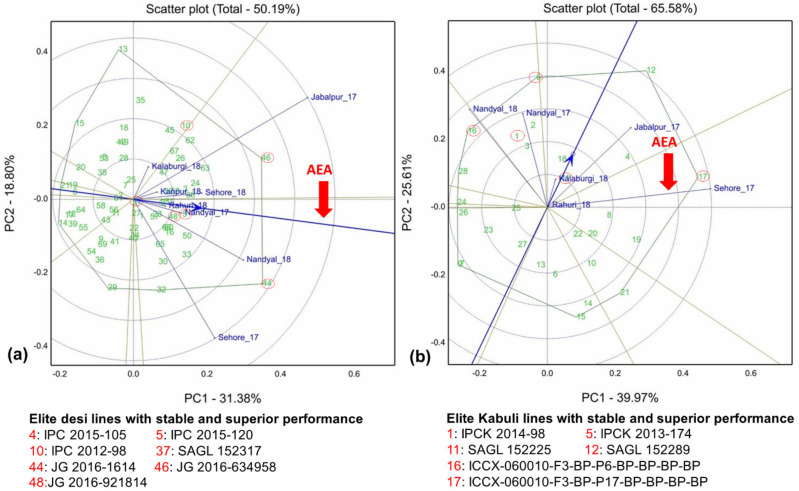
GGE biplots of (**a**) 70 desi elite lines evaluated in five locations and (**b**) 30 kabuli elite breeding lines evaluated in three locations for yield (kg/ha) during crop seasons 2016–2017 and 2018–2019. The Average Environment Axis (AEA) or Average Environment Coordination (AEC) abscissa (in blue) is the single arrowed line, which passes through the origin of the biplot and through the hypothetical average environment, denoted by the circle near (Rahuri_18 and Nandyal_17 (Desi line biplot) and Kalaburgi_18 (Kabuli lines biplot). The direction of the arrowhead on the AEA points to higher mean values for grain yield. PC1 and PC2 are the first and second principal components, respectively. Desi and kabuli lines with stable yield performance are circled in red and their genotype names are indicated below the GGE plots.

A large variation was observed in the mean performance of grain yields in elite desi and kabuli lines (including molecular breeding lines) in the states Andhra Pradesh, Karnataka, Madhya Pradesh, Maharashtra and Uttar Pradesh (Tables S3 and S4). Combined analysis of variance revealed significant differences among genotypes, environments, and genotype × environment interaction effects for grain yield in desi lines (Table 3). In the case of kabuli lines, genotype × environment and environment differed significantly. In the GGE biplot, desi and kabuli lines account for 50.18% and 65.58% of the total variation of the environment-centered genotype and GEI variation for grain yield, respectively (Figure 2a,b). The large variation among environmental means caused most of the variation in grain yield. Of 70 desi and 30 kabuli elite breeding lines, the high stability performance lines in two multi-location trials are presented in Table 4. In desi elite breeding lines across the locations in two multi-location trials, Jabalpur_2017 was identified as a highly discriminating environment. Other locations such as Nandyal_2018 and Sehore_2017 were identified as moderately discriminating environments and the rest of the environments were least discriminating for grain yield (Figure 2a). In kabuli lines, Sehore_2017 was identified as a highly discriminating environment for grain yield, while Jabalpur_2017, Nandyal_2017 and Nandyal_2018 were identified as moderately discriminating environments and Kalaburagi_2018 and Rahuri_2018 were identified as the least discriminating environments (Figure 2b).

### 3.3. Promising Desi Lines for Grain Yield Identified through Multi-Location Trials

Desi lines Phule G 0919-4-8 at Nandyal and SAGL 152210 at Sehore revealed yield advantage (6.02% and 24.15%) over the local checks across the years (Table S2). Phule G 0914-8-14 at Rahuri and IPC 2008-11 at Kanpur recorded significant yield advantage (6.42% and 7.63%) compared to the local checks (Table S2). Importantly, JG 2016-634958 at Jabalpur and SAGL 152278 at Kalaburagi demonstrated yield advantage (73.44% and 109.48%) over the local checks. Across the locations and over the years, desi line JG 2016-1614 was identified as a superior line with high grain yield and high stability and a 2.6% yield advantage over the check (Table S3).

### 3.4. Promising Kabuli Lines for Grain Yield Identified through Multi-Location Trials

Kabuli lines IPCK 2013-152 at Nandyal exhibited a yield advantage (111%) over the local check across the years (Table S3). SAGL 152220 at Kalaburagi, ICCX-060010-F3-BP-P17-BP-BP-BP-BP at Sehore, IPCK 2012-129 at Rahuri and SAGL 152289 at Jabalpur also recorded higher grain yield and yield advantage (141.29%, 21.43%, 33.25% and 116.04%, respectively) over the local checks (Table S3). A total of 30 kabuli elite breeding lines and three lines (ICCX-060010-F3-BP-P6-BP-BP-BP-BP, IPCK 2013-174 and SAGL 152289) have been identified as superior with higher grain yield and high stability and a 12.11–11.11% yield advantage over the national check (Vihar) across the locations and over the years (Table S4).

### 3.5. Enhancing Varietal Adoption through FPVS Trials

**Figure 3:**
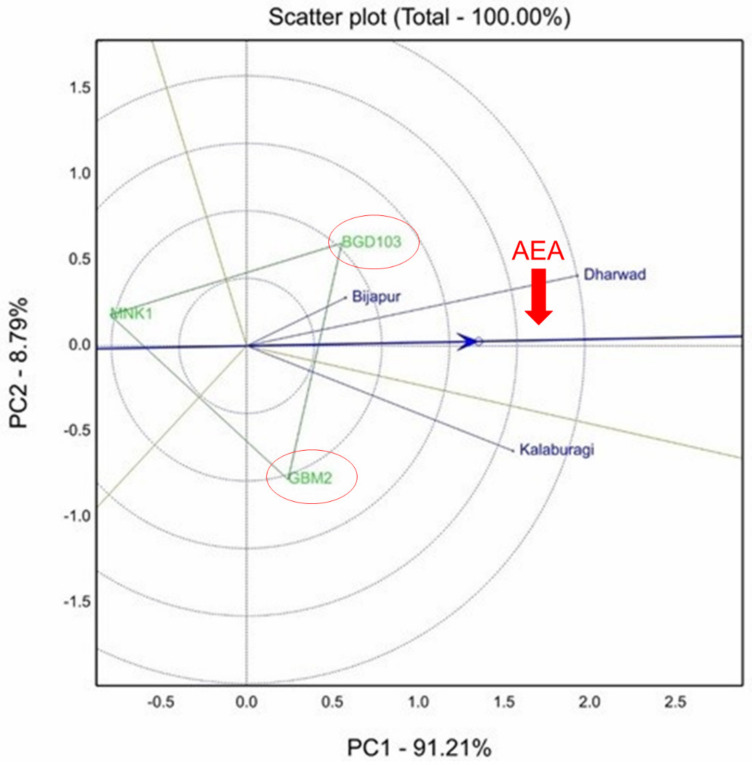
GGE biplot showing significant genotype × environment interaction (GEI) for grain yield in Karnataka during 2017–2018. Dharwad and Kalaburagi were identified as the most discriminating environments.

The mean yields of different varieties during 2017–2018, 2018–2019 and 2019–2020 are presented in Table 5. The ANOVA revealed significant differences among genotypes, but non-significant genotype × environment interaction for grain yield at all the locations over the years (Andhra Pradesh, Madhya Pradesh, Maharashtra and Uttar Pradesh) except Karnataka in 2017–2018 (Table 6). In Karnataka in 2017–2018, a GGE biplot explained stability analysis for grain yield, which accounted for 100% of the total variation of the environment-centered G × E (Table 6). Due to significant GEI, the Dharwad and Kalaburagi were identified as the most discriminating environment and significantly differed with Bijapur for grain yield (Figure 3).

### 3.6. Selection of High-Performing Varieties in Different States

**Figure 4:**
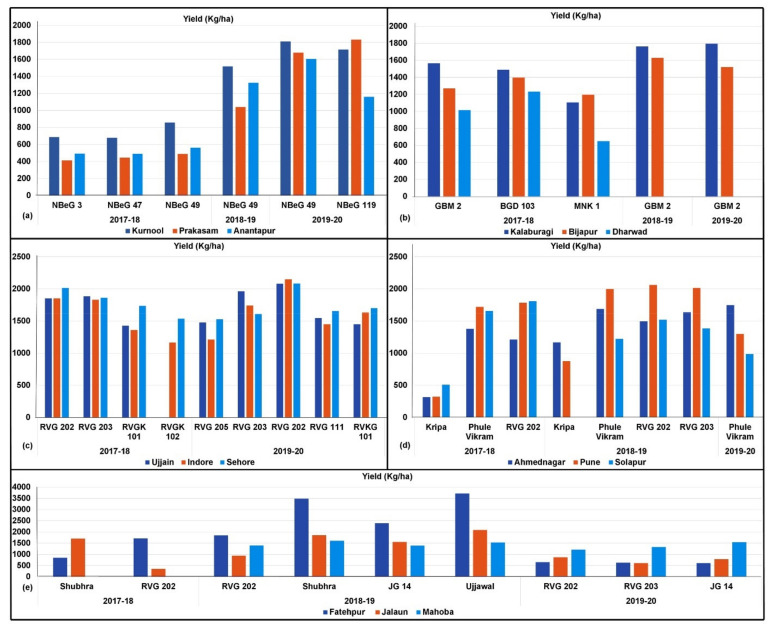
Farmer preferred varieties identified through FPVS trials conducted in different chickpea growing states. The best performing lines were (**a**) NBeG 49 in Andhra Pradesh; (**b**) GBM 2 in Karnataka; (**c**) RVG 202 in Madhya Pradesh; (**d**) Phule Vikram and RVG 202 in Maharashtra and (**e**) RVG 202 and Shubhra (IPCK 2002-29) in Uttar Pradesh.

In the FPVS trials conducted during 2017–2018 in Andhra Pradesh, NBeG 49 showed outstanding performance for grain yield and was the choice of farmers in all the three districts of Kurnool, Anantapur and Prakasam (Table 5 and Figure 4a). In the trials conducted in 2018–2019, NBeG 49 was found superior over varieties NBeG 47 and NBeG 3 tested in the region, with a 37.1% yield advantage over the local check. It is important to mention that in Kurnool district, NBeG 49 recorded a 41.9% yield advantage over the local check (Table 5). In Kurnool district, during 2019–2020, desi variety NBeG 49 and kabuli variety NBeG 119 showed 9.69% and 18.89% yield advantages, respectively over their respective local checks. Similarly, in Prakasam district, 9.37% and 13.4% yield advantage was recorded in NBeG 49 and NBeG 119, respectively. In Anantapur district, 10.77% yield advantage was recorded in NBeG 49 while it was only 1.56% in NBeG 119. Based on three years’ FPVS trials conducted in Andhra Pradesh, NBeG 49 was identified as the best performing line and was preferred by the farming community.

Based on FPVS trials conducted in Karnataka during 2017–2018, among the three elite chickpea varieties (GBM 2, BGD 103 and MNK 1), GBM 2 was found high yielding in Kalaburagi district, whereas, BGD 103 recorded high yield in Bijapur and Dharwad districts (Table 5 and Figure 4b). However, due to its traits that are amenable to mechanical harvesting, farmers from all the three districts preferred GBM 2 in 2018–2019 and 2019–2020 (Table 5 and Figure 4b).

Based on FPVS trials conducted in Madhya Pradesh during 2017–2018, RVG 202 was identified as high yielding in Indore and Sehore districts, whereas, RVG 203 was high yielding in Ujjain district (Table 5 and Figure 4c). During 2018–2019, farmers ranked the performance of different varieties. RVG 202 demonstrated superior performance in all three districts. No yield data was provided during 2018–2019 trials. In 2019–2020, FPVS trials were conducted with RVG 205, RVG 202, RVG 203, RVG 111 and RVKG 101. Of these, RVG 202 exhibited the highest grain yield of 2105 kg/ha followed by RVG 203 (1772 kg/ha) in three districts (Table 5). In Madhya Pradesh, RVG 202 followed by RVG 203 were identified as the best performing lines.

In Maharashtra, Phule Vikram was found high yielding in Ahmednagar district, whereas RVG 202 was identified as high yielding in Pune and Solapur districts in the 2017–2018 FPVS trials (Table 5 and Figure 4d). Similarly, during 2018–2019, Phule Vikram in Ahmednagar and RVG 202 in Pune and Solapur districts were identified as high-yielding varieties (Table 5 and Figure 4d). In the demonstrations in 2019–2020, the yield of Phule Vikram varied widely in all the three districts. For instance, in Ahmednagar district, it recorded the highest grain yield of 1746 kg/ha (Table 5) while yields recorded in Pune and Sholapur districts were 1278 kg/ha and 987 kg/ha yield, respectively (Table 5). Overall, Phule Vikram was widely preferred by farmers in Maharashtra. Under this study, Phule Vikram reached farmers of Pune and Solapur districts. Overall, the farmers expressed satisfaction about the varieties and showed preference for it. The productivity in Solapur district was less as compared to Ahmednagar and Pune districts due to the scarcity or nonavailability of water for irrigation during the crop seasons.

In Uttar Pradesh, during the 2017–2018 FPVS trials, variety RVG 202 showed highest grain yield in Fatehpur district and variety Shubhra was found higher yielding in Jalaun district (Table 5 and Figure 4e). While Shubhra and Ujjawal gave higher yields compared to RVG 202 in Fatehpur and Jalaun districts in 2018–2019 trials (Table 5 and Figure 4e), Shubhra recorded the highest yield in Mahoba district. In the 2019–2020 FPVS trials, RVG 202 recorded the highest grain yield in Fatehpur and Jalaun districts (Table 5) while JG 14 recorded highest grain yield of 1548 kg/ha (Table 5) in Mahoba district. RVG 202 was preferred as the best performing line by farmers in the state.

## 4. Conclusions

The TCGC has been involved in the development of traits for drought and FW associated markers and conduct of FPVS trials to enable improved varieties to reach farmers. It has successfully developed trait (biotic and abiotic stress)-associated molecular markers and is currently working on developing drought tolerance and FW resistance through the MABC approach. Multi-location and FPVS trials were used to identify high grain yield and highly stable varieties on station and in farmers’ fields. Among the desi elite breeding lines, Phule G 0919-4-8 at Nandyal, SAGL 152210 at Sehore, JG 2016-634958 at Jabalpur, SAGL 152278 at Kalaburagi, PhuleG 0914-8-14 at Rahuri and IPC 2008-11 at Kanpur revealed a high yield advantage over the local checks. Across all the locations and over the years, JG 2016-1614 was identified as a superior line with high grain yield and high stability. Likewise, kabuli elite breeding lines IPCK 2013-152 at Nandyal, SAGL 152220 at Kalaburagi, ICCX-060010-F3-BP-P17-BP-BP-BP-BP at Sehore, IPCK 2012-129 at Rahuri and SAGL 152289 at Jabalpur recorded the highest grain yields over the local checks. Across locations and over the years, ICCX-060010-F3-BP-P6-BP-BP-BP-BP followed by IPCK 2013-174 and SAGL 152289 were identified as superior lines with high grain yield and high stability. In short, NBeG 49 and GBM 2 were identified as the best performing lines preferred by farmers in Andhra Pradesh and Karnataka. RVG 202 followed by RVG 203 were the choice of farmers in Madhya Pradesh while Phule Vikram and RVG 202 were identified as the best performing lines in Maharashtra. In Uttar Pradesh, RVG 202 and Shubhra (IPCK 2002-29) were preferred by the farmers as best performing desi and kabuli lines, respectively.

The advanced drought tolerance and FW resistance backcross lines will be evaluated for grain yield for varietal release. The top performing lines and new molecular breeding lines developed in different genetic backgrounds can be further evaluated for their performance at the national level in AICRP trials on chickpea for release as improved varieties. The development of these high-yielding chickpea varieties and enhancing their adoption will increase the productivity and profitability of smallholder farmers.

## Figures and Tables

**Table 1:** The SNP panels used to select true hybrids.

Intertek ID	SNP ID	Trait	Sequence *
snpCA0001	CKAM2210	Drought	TTAAACTCACTTACCCTCTTTCCTTTCCATTTCCTTTCTTTCAAAATTCTCCTATATCCT[G/T]CTAATACAGATACTTTGCAACCCATTTTTTTTGTCAACAAAGTGTTATTGGGTGAGTTCA
snpCA0022	CKAM2227	Drought	CGAGGCCCAAATCCAAAACCGGATTCAAATTCATTTTAAATATCCGGTTAAAATCATATG[A/G]TTATAATTTGGTTTATTTATAAACCGGTTGGATAACCACTTATGTTTTATATTTGGATTT
snpCA0023	CKAM2228	Drought	CATCTGAAGATTATGTGCAGCTTAAGGTGTTGGCGGCAATTCAAGGGGACGCTAGTGTTT[C/G]TAAGGATGACAAAATTGAGCATTTGTTCTTTTCCTTAATGTTTTTTCAAAAACTCTCAAT
snpCA0004	CKAM2179	Drought	ATGTCTTCGGCTTCCAGATTTGTGTTTGGTGACATGACCGAAGAAAGCTTGAAATGAGCT[G/T]ATAGTGAAGAGCTCACTGCCTTTGATTCACACATATTGAATCTATTTAGAACCTTTCCAA
snpCA0006	CKAM2182	Drought	AACCACATGAAGAAAATAAATTATGTAAAATGTGTTGTTTCTTCGAATCAACTATGGTAT[C/T]GAGGCTATTCTGGATATCGAAGGGACATAATGAAAGAGAGAGTAGTGGCTTCGAAATGCG
snpCA0021	CKAM2226	Drought	CGCTATTAAGTACAAAAAATTGTCAAATAGCGGTTATAGCAATCTATAGCGTTGTTGCTT[A/T]GAGGAATATAAATAAACCACTATTTTTCACAATCTGCGATTCACAAAATTGGTATGTATG
snpCA0018	CKAM2223	Drought	TGAACAAAAACTTCTACGTGATCAGTTTGTCATATTTCACAAAAAAAAAAAAAAGGAATA[A/T]ATGCAATATATGCGGCTCAATTGGATGTTGTAACCATGGATTCTATTGATTAGTGGTCAA
snpCA0166	FW2_30366103	Fusarium wilt	TTCTATTATATTTTGATACTGTGGAGAATCATAGTCAAATACAATTGATA[C/A]ATACAACTTCAATTGGCCATAGAGGTCAGAGACTTCAAAAACTTTGATGT
snpCA0168	FW2_30366146	Fusarium wilt	AAATACAATTGATACATACAACTTCAATTGGCCATAGAGGTCAGAGACTT[C/A]AAAAACTTTGATGTCGCAGCTCACATCACTATCACAATCACAATCACAAT
			

* SNPs highlighted in bold are targeted loci for marker development.

**Table 2:** Summary of FPVS trials conducted during 2017–2018, 2018–2019 and 2019–2020 seasons.

State	Center	District	Variety	Number of FPVS Trials			Total FPVS Trials
				2017–2018	2018–2019	2019–2020	
Andhra Pradesh	RARS-Nandyal	Anantapur, Kurnool and Prakasam	**NBeG 47, NBeG 49 and NBeG3**	30	90	90	210
Karnataka	ARS-Kalaburagi	Bijapur, Dharwad, Gadag and Kalaburagi	**GBM 2**, BGD103, JAKI 9218 and JG 11	20	70	51	141
Madhya Pradesh	RAKCA-Sehore	Indore, Sehore and Ujjain	**RVG 202**, **RVG 203**, RVKG 101 RVKG 102, RVG 204, RVG 205 and RVKG111	30	90	90	210
Maharashtra	MPKV-Rahuri	Ahmednagar, Pune and Solapur	**Phule Vikram**, RVG 203, **RVG 202** and Kripa	30	98	76	204
Uttar Pradesh	ICAR-IIPR, Kanpur	Jalaun, Mahoba and Fatehpur	JG 14, **Ujjawal**, **Shubhra** and **RVG 202**	25	93	116	234

Varieties in bold are best performing lines preferred by farmers.

**Table 3:** Combined analysis of variance for grain yield based on multi-location trials of 100 elite lines (70 desi and 30 kabuli) conducted during cropping seasons 2016–2017 and 2018–2019.

Effect	Desi Lines	Kabuli Lines
	Variance Components	Variance Components
Environment	37.94 **	52.98 **
Replication (Environment)	0.37 **	0
Block (Environment × Replication)	0.004 *	0
Genotype	0.96 *	0.73
Environment × Genotype	8.76 **	15.93 **
Residual	13.96	25.28

* = significant at *p* < 0.05; ** = significant at *p* < 0.01.

**Table 4:** High-yielding (kg/ha) desi and kabuli lines with stable performance in multi-location trials conducted during 2016–2017 and 2018–2019.

Desi Lines during 2016–2017	Andhra Pradesh	Karnataka	Madhya Pradesh	Maharashtra
JG 2016-1614	√		√	
IPC 2012-98	√		√	
**Kabuli during 2016–2017**				
ICCX-060010-F3-BP-P17-BP-BP-BP-BP	√		√	
IPCK 2013-174	√		√	
SAGL 152225	√			
SAGL 152289			√	
**Desi lines during 2018–2019**				
IPC 2015-105			√	√
IPC 2015-120			√	√
SAGL 152317	√	√		
JG 2016-1614	√			√
JG 2016-634958	√		√	
JG 2016-921814	√		√	
**Kabuli lines during 2018–2019**				
IPCK 2014-98	√	√		
SAGL 152289	√	√		
ICCX-060010-F3-BP-P6-BP-BP-BP-BP	√			√

√ = High-yielding lines.

**Table 5:** Mean grain yield (kg/ha) of select chickpea cultivars in FPVS trials conducted in five states of India during cropping seasons 2017–2018 2018–2019 and 2019–2020.

State	Year	Variety	Kurnool	Prakasam	Anantapur	Mean
**Andhra** **Pradesh**	2017–2018	NBeG 3	686	413	492	530
		NBeG 47	679	445	491	538
		NBeG 49	857	488	561	635
	2018–2019	NBeG 49	1518	1040	1326	1295
	2019–2020	NBeG 49	1810	1679	1606	1699
		NBeG 119	1716	1833	1161	1503
**State**	**Year**	**Variety**	**Kalaburagi**	**Bijapur**	**Dharwad**	**Mean**
**Karnataka**	2017–2018	GBM 2	1565	1270	1016	1284
		BGD 103	1490	1395	1232	1372
		MNK 1	1105	1195	650	983
	2018–2019	GBM 2	1763	1628	-	1696
	2019–2020	GBM 2	1795	1521	-	1658
**State**	**Year**	**Variety**	**Ujjain**	**Indore**	**Sehore**	**Mean**
**Madhya** **Pradesh**	2017–2018	RVG 202	1854	1852	2014	1907
		RVG 203	1886	1830	1864	1860
		RVGK 101	1429	1359	1737	1508
		RVGK 102	-	1165	1537	1351
	2019–2020	RVG 205	1476	1212	1528	1405
		RVG 203	1965	1741	1610	1772
		RVG 202	2080	2150	2085	2105
		RVG 111	1548	1450	1654	1551
		RVKG 101	1450	1633	1700	1594
**State**	**Year**	**Variety**	**Ahmednagar**	**Pune**	**Solapur**	**Mean**
**Maharashtra**	2017–2018	Kripa	315	320	509	381
		Phule Vikram	1380	1720	1658	1586
		RVG 202	1211	1785	1809	1602
	2018–2019	Kripa	1169	875	-	1022
		Phule Vikram	1688	2000	1222	1637
		RVG 202	1495	2062	1521	1693
		RVG 203	1637	2015	1384	1679
	2019–2020	Phule Vikram	1746	1298	987	1344
**State**	**Year**	**Variety**	**Fatehpur**	**Jalaun**	**Mahoba**	**Mean**
**Uttar** **Pradesh**	2017–2018	Shubhra	850	1700	-	1275
		RVG 202	1715	350	-	1033
	2018–2019	RVG 202	1846	938	1401	1395
		Shubhra	3484	1853	1608	2315
		JG 14	2388	1556	1392	1779
		Ujjawal	3718	2085	1529	2444
	2019–2020	RVG 202	650	869	1209	909
		RVG 203	629	610	1325	855
		JG 14	608	786	1548	980

**Table 6:** Combined analysis of variance (F-value) for grain yield based on FPVS trials in centers in the respective states.

Effect	Andhra Pradesh (Nandyal) 2017–2018	Karnataka (Kalaburagi) 2017–2018	Madhya Pradesh (Sehore) 2017–2018	Maharashtra (Rahuri) 2017–2018	Maharashtra (Rahuri) 2018–2019	Uttar Pradesh (Kanpur) 2017–2018	Uttar Pradesh (Kanpur) 2018–2019
District	2.1	9.50 **	2.02	0.81	0.79	3.3	6.77 **
Variety	7.26 **	89.79 **	27.47 **	0.08	0.02	3.67	0.26
District × Variety	1.75	36.26 **	2	0.59	0.21	1.29	0.29
Residual	0.25	0.52	1.57	10.78	7.47	14.87	14.98

** = significant at *p* < 0.01.

## Data Availability

Not applicable.

## Supplementary Materials

The following are available online at https://www.mdpi.com/article/10.3390/plants10122583/s1, Table S1: Details of allele specific markers for Fusarium wilt; Table S2: Details of marker-assisted backcrossing to improve elite lines for drought tolerance and resistance to fusarium wilt, Table S3: Mean performance of 70 elite desi lines evaluated in different locations during 2016–2017 and 2018–2019, Table S4: Mean performance of 30 elite kabuli lines evaluated in different locations during 2016–2017 and 2018–2019.
